# Identification of Prognostic Markers in Patients with Primary Vitreoretinal Lymphoma by Clustering Analysis Using Clinical Data

**DOI:** 10.3390/jcm9072298

**Published:** 2020-07-20

**Authors:** Kinya Tsubota, Yoshihiko Usui, Hiroshi Goto

**Affiliations:** Department of Ophthalmology, Tokyo Medical University, 6-7-1 Nishishinjuku, Shinjuku-ku, Tokyo 160-0023, Japan; usuyoshi@gmail.com (Y.U.); goto1115@tokyo-med.ac.jp (H.G.)

**Keywords:** primary vitreoretinal lymphoma, prognostic biomarker, clustering analysis, uveitis

## Abstract

(1) Purpose: Primary vitreoretinal lymphoma (PVRL) is associated with poor prognosis because most of the patients with PVRL develop central nerve system lymphoma. The prognostic biomarker of PVRL is largely unknown. Cluster analysis has been used to identify phenotypic groups within various diseases. In this study, we aimed to describe clinical features of patients with PVRL grouped by clustering analysis and to identify biomarkers for predicting survival prognosis in patients with PVRL. (2) Materials and Methods: Forty patients with PVRL were divided into two groups by clustering analysis using clinical data. Clinical features of the two groups were compared. (3) Result: Clustering analysis classified patients into groups A and B. The survival rate during the follow-up period was significantly lower in group B than in group A (*p* = 0.03). Serum IgG, serum IgA, vitreous IL-10 and vitreous IL-10 to IL-6 ratio were significantly different between groups A and B (*p* = 0.03, 0.005, 0.008 and 0.03, respectively). Receiver operating characteristic (ROC) curves generated for the four variables indicated that serum IgA was most suitable for the prediction of prognosis. Patients with serum IgA below 184 mg/dL obtained from the ROC curve had a lower three-year survival rate (*p* = 0.03) and more episodes of recurrence of lymphoma (3.2 times versus 1.8 times, *p* = 0.02) compared with patients with serum IgA above 184 mg/dL. (4) Conclusion: The survival rate was significantly different in PVRL patients classified into two groups by clustering analysis. Patients with lower serum IgA had more recurrences and poorer survival than patients with higher IgA.

## 1. Introduction

Primary vitreoretinal lymphoma (PVRL), previously known as primary intraocular lymphoma (PIOL), is a form of non-Hodgkin’s lymphoma of the diffuse large B cell type (DLBCL) and constitutes approximately less than 1% of all intraocular tumors [1]. PVRL is associated with poor prognosis because most of the patients with PVRL develop central nerve system lymphoma (CNSL) within a few years after onset of PVRL. The five-year survival rate of PVRL is approximately 30–60% [2,3]. PVRL is often misdiagnosed as uveitis at disease onset because PVRL is a masquerade syndrome that manifests features of uveitis including vitreous opacity, subretinal lesion, keratic precipitates and iritis [3,4,5]. Misdiagnosis of PIOL at onset increases the risk of missed or delayed diagnosis of CNSL [6,7,8,9]. For this reason, PVRL is diagnosed for the first time after onset of CNSL in some patients. Currently, there is no gold standard for diagnosing PVRL, which makes diagnosis difficult. A diagnosis of PVRL requires a comprehensive assessment of multiple examinations. Cytology using vitreous humor, measurement of cytokine levels (IL-10 to IL-6 ratio), evaluation of immunoglobulin heavy chain (IgH) gene rearrangement and analysis of cell surface markers using flow cytometry are useful for the diagnosis of PIOL [10]. Local treatment for an ocular lesion consists of radiation or methotrexate injection. On the other hand, radiation of whole brain or systemic chemotherapy is needed with the development of CNSL. Our previous multicenter study in Japan which analyzed the clinical course of PIOL found that 60% of the patients had both PIOL and CNSL lesions and that the five-year survival rate was 61% [3]. 

Patients’ general conditions deteriorate gradually after onset of CNSL, if patients survive for more than five years. We previously analyzed the clinical features of 14 patients who were diagnosed with PIOL at the Department of Ophthalmology of the Tokyo Medical University and followed for over five years [11]. The outcome of the disease varied: some patients did not develop CNSL, while others had more severe disease. Indeed, information based on prognostic biomarkers identified in PVRL patients may impact clinical decisions, and potentially add to the understanding of why some patients with CNSL develop severe disease, whereas others do not. Therefore, the identification of appropriate prognostic biomarkers for PVRL that are linked to clinical features is important. However, no attempt has been made thus far to determine the prognostic biomarkers based on clinical features of PVRL. Further, no biomarker for the prediction of survival has demonstrated sufficient sensitivity or specificity.

Cluster analysis refers to statistical methods that attempt to discriminate relatively homogeneous groups of patients based on selected characteristics. Recently, cluster analysis has been used to identify phenotypic groups within various diseases [12,13,14,15]. In this study, we sought to identify prognostic biomarkers by grouping PVRL patients by clustering analysis using clinical data and comparing the clinical features including survival rate and recurrences between the groups.

## 2. Materials and Methods

We conducted a retrospective, non-interventional single-institutional observational study. This study was conducted in compliance with the Helsinki principles. Ethical approval was obtained from the Medical Ethical Research Committee in Tokyo Medical University Hospital. All patients signed an informed consent form before participation.

### 2.1. Patients

One hundred and twenty-four patients diagnosed with PVRL at the Department of Ophthalmology, Tokyo Medical University Hospital between April 1999 and December 2019 were identified. Among the 124 patients, 60 patients were excluded because the follow-up period was less than 3 years, and 24 patients were excluded because the appropriate peripheral blood test data used in analysis, including IgG and IgA levels, were not available. The remaining 40 patients with PVRL were included in this analysis. The subjects consisted of 17 males and 23 females, with an average age at onset of 65.7 ± 9.7 years. All patients were Asian adults. All patients underwent magnetic resonance imaging (MRI) to evaluate the brain, as well as a standard 3-port 25-gauge (5000 cycle/min, CONSTELLATION^®^, Alcon, Fort Worth, TX, USA), 23-gauge or 20-gauge (2500 cycle/min, ACCURUS^®^, Alcon, Fort Worth, TX, USA) vitrectomy. Peripheral blood tests were performed at the first visit to the hospital. The diagnosis of PVRL was established based on clinical, morphologic, cytochemical, gene rearrangement and immunologic features. A mid-vitreous sample was collected with a vitreous cutter at the start of vitrectomy before intraocular infusion. Cytopathologic and immunocytochemical evaluations were performed on an undiluted vitreous specimen to evaluate features of the lymphoma. The concentration of cytokine, status of IgH chain gene rearrangement and cell surface markers in the vitreous humor sample were assayed using an enzyme-linked immunosorbent assay, polymerase chain reaction and flow cytometer at a commercial laboratory (SRL Inc., Tokyo, Japan). 

The first treatment was intravitreal injection of methotrexate when the original lesion involved a unilateral eye, radiation therapy when the original lesion involved bilateral eyes and systemic chemotherapy when the original lesion was in the central nerve system (CNS). Recurrence in a unilateral eye was treated with intravitreal methotrexate injection, and recurrence in bilateral eyes was treated with radiation therapy when the patient had not received radiation therapy before recurrence. Recurrence in CNS or other tissues or organs excluding the eye (such as lymph node) was treated with systemic chemotherapy. Prophylactic systemic chemotherapy was not given to patients without CNSL because a multicenter analysis in Europe indicated no improvement of survival by systemic chemotherapy in patients with PVRL who did not develop CNSL [16]. The following clinical data were extracted from the medical record of each patient: sex, age at diagnosis of PVRL, follow-up period, onset of bilateral or unilateral disease, original lesion site, presence of CNS involvement, cytopathology, status of IgH chain gene rearrangement, concentrations of cytokines in vitreous fluid, results of peripheral blood tests (white blood cell count, soluble IL-2 receptor, beta2-microglobulin, immunoglobulins G and A), number of recurrences after first diagnosis of PVRL, progression-free survival period and survival rate. 

### 2.2. Clustering Analysis

Hierarchical cluster analysis was performed using the following continuous variables: age at onset, peripheral white blood cell (WBC) count, soluble IL-2 receptor (sIL-2), beta2-microglobulin (β2MG), immunoglobulin G (IgG), immunoglobulin A (IgA), concentrations of IL-10 and IL-6 in vitreous fluid and ratio of IL-10 to IL-6 at diagnosis of PVRL. Analysis was performed using statistical software. We divided the patients into two groups by clustering analysis. We then compared the 2 groups divided by clustering analysis for the following variables: clinical features, sex, follow-up period, survival rate, number of recurrences, progression-free survival, cytological analysis, frequency of IgH gene rearrangements, peripheral blood tests and cytokines in vitreous humor. 

### 2.3. Statistical Analysis

A chi-square test was used to compare clinical features, peripheral blood tests and concentrations of cytokines in vitreous humor between the two groups divided by clustering analysis. Receiver operating characteristic (ROC) curves were plotted for serum IgG, serum IgA, vitreous IL-10 and vitreous IL-6/IL-10 ratio. Kaplan–Meier survival curves were generated for patients divided by cutoff serum IgA level obtained from the ROC curve. A log-rank test was used to assess 5-year survival. A *p* value less than 0.05 was considered statistically significant. All analyses were performed using commercial statistical analysis software (BellCurve for Excel, Social Survey Research Information Co. Ltd., Japan; JMP, SAS Institute, Ltd., Japan).

## 3. Results

### 3.1. Patient Demographics, Lesion Laterality, Site of Origin Lesion and Treatment

Patient demographics are shown in Table 1. The 40 patients consisted of 17 males and 23 females, with an average age at onset of 65.7 ± 9.7 (mean ± SD) years. Average follow-up period was 43.2 ± 24.2 months. Twenty-one cases (53%) had bilateral onset, and 19 cases (47%) had unilateral onset. The original lesion was intraocular only without CNS involvement in 34 cases (85%), both intraocular and CNS in 1 case (2%) and only CNS without intraocular involvement in 5 cases (13%). Treatment for the original lesion was radiation in 15 cases (38%), intravitreal injection of methotrexate in 20 cases (50%) and systemic chemotherapy in 6 cases (15%).

### 3.2. Medical Examinations and Clinical Courses in All Patients with PVRL

Medical examinations including cytological grades, status of IgH gene rearrangement, vitreous IL-10 and IL-6 concentrations and peripheral blood test data are shown in Table 2. For cytological grading, 20 cases (50%) were below class IIIa, 8 cases (20%) were class IIIb, 7 cases (18%) were above class IV and 5 cases (12%) were unclassified. IgH gene rearrangement was present in 14 cases, absent in 8 cases and not tested in 18 cases (45%) due to insufficient samples. For cytokines in the vitreous humor, the average concentration of IL-10 was 3247.2 ± 5541.8 pg/mL, average concentration of IL-6 was 86.3 ± 133.0 pg/mL and 32 cases (80%) had IL-10/IL-6 ratios higher than 1. The average WBC count was 6642.6 ± 2425.1/μL, average serum sIL-2 level was 374.9 ± 257.5 pg/mL, average serum β2MG level was 1.5 ± 0.6 mg/dL and average IgG and IgA levels were 1241.1 ± 492.5 and 198.2 ± 83.9 mg/dL, respectively. 

The clinical courses are summarized in Table 3. The average number of recurrences was 2.5 ± 1.7 for all sites, 1.4 ± 1.4 in the intraocular site, 1.1 ± 0.9 in the CNS and 0.2 ± 0.6 in other sites. The overall progression-free survival was 15.6 ± 9.4 months. Twenty-eight patients (70%) were alive and 12 patients (30%) died during the follow-up period. 

### 3.3. The Differences of Clinical Features in Two Groups Classified according to Clustering Analysis

**Figure 1:**
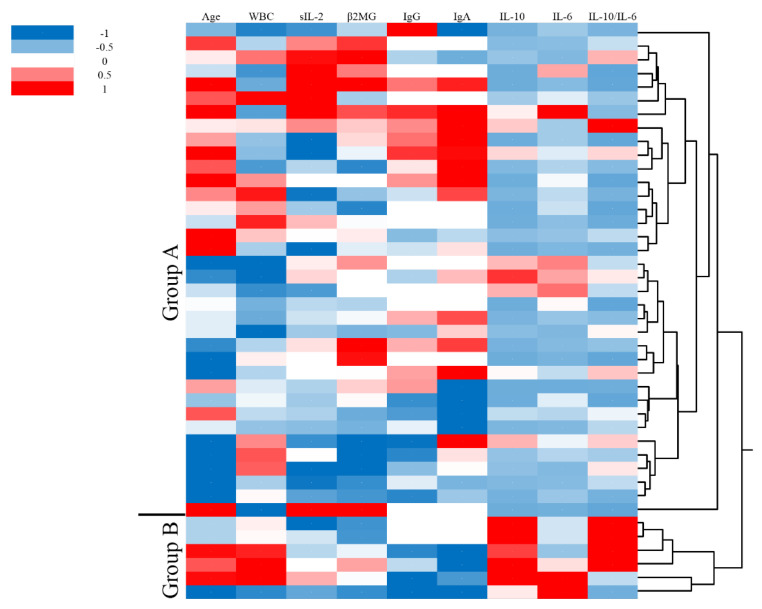
The patients were clustered into two groups by clustering analysis using clinical findings including age, peripheral blood tests and cytokines in vitreous humor.

Clustering analysis is shown in Figure 1. Using unbiased clustering analysis, patients were divided into two groups. Group A consisted of patients who had low vitreous IL-10, high serum IgA and low WBC count. Group B consisted of patients who had high vitreous IL-10, low serum IgA and high WBC count. 

We then compared the clinical features and laboratory findings between groups A and B. Clinical features in the two groups are shown in Table 4. The survival rate during the follow-up period was significantly poorer in group B than in group A (*p* = 0.03). Male/female ratio, average age, average follow-up period, number of recurrences, progression-free survival, proportion of cytological grade above class IV, frequency of IgH gene rearrangement and first treatment were not significant different between the two groups. Laboratory findings for the two groups are shown in Table 5. Serum IgG, serum IgA, vitreous IL-10 and vitreous IL-10/IL-6 ratio were significantly different between the two groups (*p* = 0.03, 0.005, 0.008 and 0.03, respectively). Average age, WBC count, serum sIL-2, serum β2MG and vitreous IL-6 were not different between the two groups. These results indicated that patients with poorer prognosis had lower serum IgG, lower serum IgA, higher vitreous IL-10 and a higher vitreous IL-10/IL-6 ratio. Therefore, prognosis can be predicted by laboratory findings.

### 3.4. ROC Curves for 4 Findings of Medical Examinations Showing Differences between Two Groups

**Figure 2:**
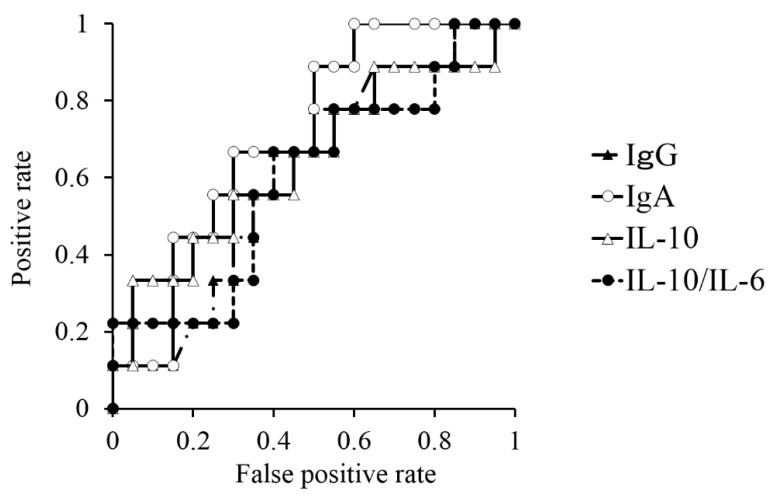
Receiver operating characteristic (ROC) curves for serum IgG, serum IgA, vitreous IL-10 and vitreous IL-10/IL-6 ratio. The area under the curve (AUC) of IgA is the biggest among the four markers. Cutoff value of IgA is 184 mg/dL, with the highest odds ratio.

We plotted ROC curves to investigate which laboratory finding among IgG, IgA, IL-10 and IL-10/IL-6 ratio is most suitable for predicting prognosis. ROC curves for IgG, IgA, IL-10 and IL-10/IL-6 ratio are shown in Figure 2 and Table 6. The areas under the ROC curves for IgG, IgA, IL-10 and IL-10/IL-6 ratio were 0.61, 0.71, 0.64 and 0.60, respectively. This result indicates that serum IgA is most suitable for the prediction of prognosis.

### 3.5. Differences in Clinical Features between Patients Divided by Cut-Off Serum IgA Concentration of 184 mg/dL

**Figure 3:**
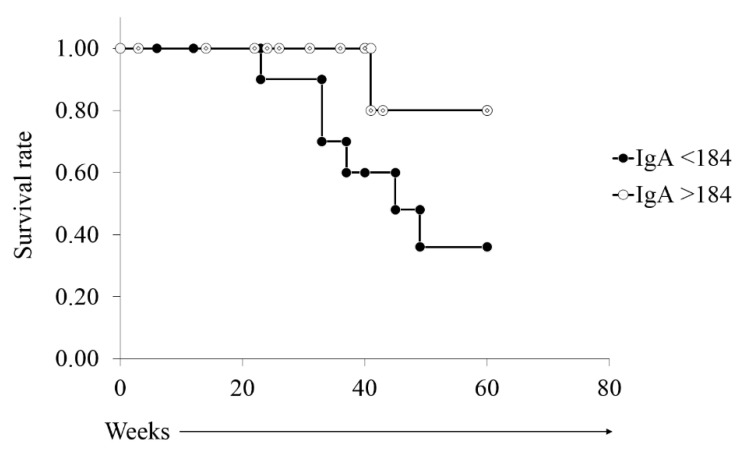
Kaplan–Meier plots of patients stratified by serum IgA of 184 mg/dL. Patients with serum IgA below184 mg/dL had worse survival than patients with IgA above 184 mg/dL (log-rank test, *p* = 0.06).

Next, we analyzed survival by the Kaplan–Meier method in patients stratified by the cutoff serum IgA concentration obtained from the ROC analysis. Patients with IgA below 184 mg/dL tended to have poorer prognosis of survival in a five-year period, although the difference was not statistically significant (*p* = 0.06) (Figure 3). When we compared the clinical features of patients divided by the serum IgA concentration of 184 mg/dL (Table 7), patients with IgA below 184 mg/dL had more recurrences than patients with IgA above 184 mg/dL (3.2 versus 1.7, *p* = 0.02). Moreover, the three-year survival rate was significantly worse in patients with IgA below 184 mg/dL compared with those with IgA above 184 mg/dL (*p* = 0.03). These results indicate that patients with serum IgA lower 184 mg/dL have poor prognosis due to frequent recurrences. 

## 4. Discussion 

In 1987, Freeman et al. [2] reported that the five-year survival rate of PIOL (formally termed PVRL) was lower than 30%. In 2012, however, a multicenter study of PIOL in Japan reported a five-year survival rate of 61.1% [3]. Recently, five-year survival rates of 54.4% to 55.8% in patients with PIOL have been reported in some countries [17,18]. This improvement of survival may be attributed to early detection, early diagnosis and extension of systemic chemotherapy using methotrexate. However, a multicenter analysis in Europe indicated that improvement of survival by systemic chemotherapy was not observed in patients with PVRL who did not develop CNSL [16]. Moreover, the European multicenter showed that development of CNSL alone decreased survival [16]. Therefore, the reason for recent improvements of survival may be attributed to the early initiation of treatment after onset of CNSL because different treatments do not affect survival. Indeed, some patients with PVRL die due to recurrence in organs or tissues that possess functions vital for survival, and repeated CNS recurrence resulting in the loss of CNS functions. For these reasons, predicting the development and severity of CNSL is very important. To date, there are no validated biomarkers for predicting the severity and/or disease outcome in PVRL patients.

Recently, some articles have reported that the ratios of different types of peripheral blood cells, such as the lymphocyte-to-monocyte ratio (LMR) and neutrophil-to-lymphocyte ratio (NLR), may be prognostic markers for malignant tumors including lymphoma [19,20,21,22,23]. Patients with DLBCL who have high NLR are more likely to have poorer prognosis than patients with lower NLR [23]. NLR is one of the biomarkers for systemic inflammation. In other words, patients with high-grade systemic inflammation have poor prognosis. Patients with low serum IgG and IgA may have significant systemic inflammation due to an immunocompromised condition because serum IgG and IgA play important roles in immunity against infections. Recurrent infections such as sinusitis, bronchitis and pneumonia are the most common infections seen in patients with selective IgA deficiency [24]. The causes of death in PVRL patients with long survival are not directly lymphoma-related, but are indirectly related, such as aspiration pneumonia due to declined activities of daily living as a result of frequent recurrences. Therefore, low serum IgG and IgA may be prognostic markers in patients with lymphoma.

Yang et al. [25] reported that STAT3 activation is associated with IL-10 expression and prognosis of CNSL. IL-10 is one of the prognostic markers of CNSL. An increased IL-10/IL-6 ratio is a useful marker for the diagnosis of PVRL [8,26,27]. IL-10 has an immunosuppressive effect by inhibiting the production of Th1-associated cytokines and inhibiting the proliferation of B cells [28]. Chan et al. [9] also reported that a concentration of IL-10 in the vitreous was associated with the number of malignant cells in vitreous humors. Therefore, these reports indicate that the prognosis of PIOL may be predicted by the concentration of IL-10 in vitreous humors [29]. In this study, concentrations of IL-10 and IL-10/IL-6 ratio in the vitreous humor were higher in patients with poorer prognosis compared with those with better prognosis. Therefore, the addition of vitreous IL-10 concentration and/or IL-10/IL-6 ratio that predict prognosis and extraocular involvement may further increase the confidence of delivering more personalized management for PVRL, particularly for those with CNSL. 

Immunoglobulins are produced by B cells. Immunoglobulins are reduced when normal lymphocytes are suppressed due to proliferation of abnormal lymphocytes. In fact, decreases in serum IgA and IgG are associated with progression of disease stage in malignant lymphoma and chronic lymphocytic leukemia [30]. Hence, serum IgA and IgG may also decrease in association with progression of PVRL. A recent study reported that 60–80% of patients with PVRL have the mutation of *MYD88* [31,32]. The mutation of *MYD88* is a prognostic marker of primary CNSL [33]. Therefore, the *MYD88* mutation may also be a prognostic marker of PVRL. IgA^+^ plasmablasts in the intestinal duct were decreased in *MYD88* knock-out mice [31]. Therefore, the *MYD88* mutation may be involved in the production of IgA. These reports suggest that decreased serum IgA may be associated with prognosis because low IgA might be related to the *MYD88* mutation in patients with PVRL.

In this study, survival was significantly different in patients with PVRL classified into two groups based on the result obtained from the clustering analysis using clinical data. The ultimate goal of this classification is to improve the prediction of a patient’s risk of the development of CNSL, so as to aid the ophthalmologist/hematologist in the decision-making on follow-up and treatment strategy (such as whether a patient should undergo brain MRI). Serum IgG, serum IgA, vitreous IL-10 and vitreous IL-10/IL-6 ratio were all significantly different between the two groups. Serum IgA had the biggest AUC among the four markers (IgG, IgA, IL-10 and IL-10/IL-6 ratio). Patients with lower serum IgA had more episodes of recurrence than patients with higher serum IgA. On the other hand, the five-year survival rate tended to be different between patients with lower IgA and those with higher IgA, but the difference did not reach statistical significance (*p* = 0.06). This may be due to the small sample size. Further study on a larger number of patients is needed to improve the statistical power. Moreover, all patients did not receive the same treatment due to the retrospective nature of this study. A prospective study is required to verify the present results. Nevertheless, this study demonstrates that prognosis can be predicted from analyses of clinical data. Indeed, recommendations based on serum IgA, which is commonly measured as a clinical laboratory test in PVRL patients, may impact clinical decisions, and potentially add to the understanding of why some PVRL patients develop CNSL while others do not. Providing personalized medicine to patients remains a challenge, but this is the roadmap by which appropriate examinations such as brain MRI will be delivered to the right patient.

We recognize several strengths and weaknesses of this study. This is the first study to use unsupervised cluster analysis of a panel of biomarkers in patients with PVRL. This study demonstrates the potential of using a group of commonly available laboratory test data in the prediction of prognosis of PVRL patients, which will have wide applicability once validated. On the other hand, there are several limitations. First, the retrospective nature of the study may cause selection bias and confounding bias. The sample size was small because of the rarity of the disease, the difficulty of diagnosing PVRL due to the absence of a gold standard and the lack of more detailed clinical information. A larger number of patients should be examined in a multicenter study. Second, because all cases were collected from a single tertiary care hospital, there is potential referral bias. Future studies will need to prospectively validate these biomarkers in independent cohorts and evaluate whether the addition of other markers could improve the accuracy of predicting the prognosis of PVRL. Furthermore, future studies could assess whether this panel can predict extraocular lesions such as CNSL.

Recently, omics analysis is mainstream for the search of prognostic biomarkers. The four clinical biomarkers (serum IgG, serum IgA, vitreous IL-10 and vitreous IL-10/IL-6 ratio) identified in this study will be further boosted by the integration of genetic, transcriptomic, proteomic and metabolomics technologies to define the prognosis of PVRL phenotypes. This will pave the way towards personalized medicine and stratified care for PVRL. Our findings contribute to increase the knowledge of new biomarkers that can potentially predict extraocular lesions and prognosis of PVRL in the future. 

## Figures and Tables

**Table 1:** Patient demographics, lesion laterality at onset, origin lesion and treatment.

Characteristics	No. (%) or Average (±SD)
Number of patients	40
Gender: male/female	17/23 (43%/58%)
Age (years)	65.7 ± 9.7
Follow-up period (months)	43.2 ± 24.2
Onset: bilateral/unilateral	21/19 (53%/47%)
Origin lesion	
Intraocular	34 (85%)
CNS	5 (13%)
Intraocular and CNS	1 (2%)
Treatment	
Radiation	15 (38%)
MTX-IVI	20 (50%)
Chemotherapy	6 (15%)

CNS = central nerve system; MTX = methotrexate; IVI = intravitreal injection.

**Table 2:** Medical tests: cytological analysis and others.

Test	No. (%) or Average (±SD)
Cytological analysis	
Below class IIIa	20 (50%)
Class IIIb	8 (20%)
Above class IV	7 (18%)
Not classified	5 (12%)
Incidence of IgH gene rearrangements	
Present	14 (35%)
Absent	8 (20%)
Not tested	18 (45%)
Cytokine in vitreous humor	
IL-10 (pg/mL)	3247.2 ± 5541.8
IL-6 (pg/mL)	86.3 ± 133.0
IL-10/IL-6 over 1	32 (80%)
Peripheral blood test	
WBC (number/μL)	6642.6 ± 2425.1
sIL-2 (pg/mL)	374.9 ± 257.5
β2MG (mg/dL)	1.5 ± 0.6
IgG (mg/dL)	1241.1 ± 492.5
IgA (mg/dL)	198.2 ± 83.9

IgH = immunoglobulin heavy chain; IL-10 = interleukin 10; IL-6 = interleukin 6; WBC = white blood cell; sIL-2 = soluble interleukin 2 receptor; β2MG = beta 2 micro globulin; IgG = immunoglobulin G; IgA = immunoglobulin A.

**Table 3:** Clinical course: recurrences and others in follow-up period.

Characteristics	No. (%) or Average (±SD)
Number of recurrences (times)	2.5 ± 1.7
Intraocular	1.4 ± 1.4
CNS	1.1 ± 0.9
Other	0.2 ± 0.6
Progression-free survival (months)	15.6 ± 9.4
Survival rate (%)	
Alive	28 (70%)
Dead	12 (30%)

CNS = central nerve system.

**Table 4:** Clinical features and treatment in the two groups, classified based on clustering analysis.

Characteristics	Group A (*n* = 34)	Group B (*n* = 6)	*p* Value
Gender: male/female	15/19	2/4	0.62
Age (years)	65.6	66.3	0.87
Follow-up period (months)	44.1	38.2	0.59
Cytological analysis			
Above class IV (%)	15	33	0.27
IgH gene rearrangements			
Present (%)	33	33	0.92
Number of recurrences	2.6	2.7	0.93
Intraocular	1.4	1.2	0.64
CNS	1.1	1.3	0.62
Other	0.3	0.2	0.67
Progression-free survival (months)	14.6	18.8	0.61
Survival rate (%)	65	17	0.03
Treatment			
Radiation	12 (35%)	3 (50%)	0.49
MTX-IVI	19 (56%)	1 (17%)	0.07
Chemotherapy	4 (12%)	2 (33%)	0.17

IgH = immunoglobulin heavy chain; CNS = central nerve system; MTX = methotrexate; IVI = intravitreal injection.

**Table 5:** Laboratory findings for the two groups, classified based on clustering analysis.

Characteristics	Group A (*n* = 34)	Group B (*n* = 6)	*p* Value
Age (years)	65.6		0.88
WBC (number/μL)	6194		0.15
sIL-2 (pg/mL)	345	275	0.29
β2MG (mg/dL)	1.39	1.27	0.32
IgG (mg/dL)	1271	875	0.03
IgA (mg/dL)	222	114	0.005
Cytokine in vitreous humor			
IL-10 (pg/mL)	1238	12,767	0.008
IL-6 (pg/mL)	71	335	0.17
IL-10/IL-6 over than 1	19	1341	0.03

WBC = white blood cell; sIL-2 = soluble interleukin 2 receptor; β2MG = beta 2 micro globulin; IgG = immunoglobulin G; IgA = immunoglobulin A; IL-10 = interleukin 10; IL-6 = interleukin 6.

**Table 6:** Cutoff value obtained from the ROC curve.

Markers	AUC	Odds Ratio	Cut off Value	
IgG	0.61	3	1187	mg/dL
IgA	0.71	4.67	184	mg/dL
IL-10	0.64	2.91	1630	pg/dL
IL-10/IL-6	0.6	3	20.45	

AUC = area under ROC curve; IgG = immunoglobulin G; IgA = immunoglobulin A;.IL-10 = interleukin 10; IL-6 = interleukin 6.

**Table 7:** Clinical findings in patients with IgA lower and higher than 184 mg/dL.

Characteristics	Less than 184 mg/dL	High than 184 mg/dL	*p* Value
Age (years)	64.4	67.3	0.46
Follow-up period (months)	41.4	32.7	0.31
Cytological analysis			
Above class IV (%)	17	17	0.61
Incidence of IgH gene rearrangements			
Present (%)	50	28	0.21
Number of recurrences	3.2	1.8	0.02
Intraocular	1.3	1.2	0.84
CNS	1.3	0.9	0.4
Other	0.7	0	
Progression-free survival (months)	29.5	19	0.15
Three-year survival rate (%)	67	100	0.03

IgA = immunoglobulin A; CNS = central nerve system.
